# Analysis of Determinants in Filovirus Glycoproteins Required for Tetherin Antagonism

**DOI:** 10.3390/v6041654

**Published:** 2014-04-09

**Authors:** Kerstin Gnirß, Marie Fiedler, Annika Krämer-Kühl, Sebastian Bolduan, Eva Mittler, Stephan Becker, Michael Schindler, Stefan Pöhlmann

**Affiliations:** 1 Infection Biology Unit, German Primate Center, 37077 Göttingen, Germany; E-Mails: KGnirss@dpz.eu (K.G.); MFiedler@dpz.eu (M.F.); annika.kraemer-kuehl@boehringer-ingelheim.com (A.K.-K.); 2 Institute of Virology, Hannover Medical School, 30625 Hannover, Germany; 3 Institute of Virology, Helmholtz Center Munich, 85764 Neuherberg, Germany; E-Mails: sebastian.bolduan@helmholtz-muenchen.de (S.B.); michael.schindler@helmholtz-muenchen.de (M.S.); 4 Institute of Virology, Philipps-University-Marburg, 35043 Marburg, Germany; E-Mails: mittlere@staff.uni-marburg.de (E.M.); becker@staff.uni-marburg.de (S.B.)

**Keywords:** tetherin, ebola, lassa, glycoprotein

## Abstract

The host cell protein tetherin can restrict the release of enveloped viruses from infected cells. The HIV-1 protein Vpu counteracts tetherin by removing it from the site of viral budding, the plasma membrane, and this process depends on specific interactions between the transmembrane domains of Vpu and tetherin. In contrast, the glycoproteins (GPs) of two filoviruses, Ebola and Marburg virus, antagonize tetherin without reducing surface expression, and the domains in GP required for tetherin counteraction are unknown. Here, we show that filovirus GPs depend on the presence of their authentic transmembrane domains for virus-cell fusion and tetherin antagonism. However, conserved residues within the transmembrane domain were dispensable for membrane fusion and tetherin counteraction. Moreover, the insertion of the transmembrane domain into a heterologous viral GP, Lassa virus GPC, was not sufficient to confer tetherin antagonism to the recipient. Finally, mutation of conserved residues within the fusion peptide of Ebola virus GP inhibited virus-cell fusion but did not ablate tetherin counteraction, indicating that the fusion peptide and the ability of GP to drive host cell entry are not required for tetherin counteraction. These results suggest that the transmembrane domains of filoviral GPs contribute to tetherin antagonism but are not the sole determinants.

## 1. Introduction

Research conducted in the last 15 years revealed that host cells can express antiviral effector molecules, which inhibit spread of certain viruses by interfering with discrete steps in the viral life cycle [1,2]. The expression of these virus-restricting factors, short restriction factors, can be constitutive but is frequently inducible by interferon (IFN). Single restriction factors have been identified by screening approaches seeking to determine the molecular mechanism underlying a specific antiviral activity [3,4,5,6,7]. A recent systematic screen for IFN-induced antiviral effectors provided detailed insights into the full restriction factor repertoire of the host cell [8]. One of the restriction factors uncovered in this screen [8] and previously identified as a cellular protein which inhibits release of HIV-1 [5] was the transmembrane protein tetherin (BST-2, CD317). It is now appreciated that tetherin restricts the release of several enveloped viruses and recent findings suggest a significant contribution of tetherin to retrovirus control in experimentally infected mice [9,10].

Tetherin’s unusual membrane topology is the basis for its antiviral activity. Tetherin contains two membrane anchors, a transmembrane domain at the N-terminus and a C-terminal GPI anchor [11]. It has been proposed that the latter might meet the criteria of a transmembrane domain more than those of a GPI anchor [12]. As a consequence of the two membrane-anchoring domains, tetherin can simultaneously insert into the plasma membrane and the viral membrane, thereby forming a physical connector between host cell and virus, which prevents release of progeny particles into the extracellular space [13,14]. The importance of tetherin’s domain organization for its antiviral activity is highlighted by two observations: Mutation of either membrane-anchoring domain interferes with inhibition of viral release and artificial tetherin molecules, which bear no sequence similarity with wt tetherin but exhibit the same domain organization, display antiviral activity [13].

In order to ensure efficient spread in tetherin positive cells, several viruses encode proteins which antagonize the antiviral activity of tetherin, mainly by removing tetherin from the site of viral budding. The first tetherin antagonist identified was the HIV-1 accessory protein Vpu [5]. Vpu antagonizes tetherin by removing it from the plasma membrane [15]. This process depends on interactions between the transmembrane domains of Vpu and tetherin [16,17,18,19], which allow for a Vpu-dependent alteration of tetherin trafficking. Thus, Vpu interactions with tetherin prevent transport of newly synthesized or recycled tetherin to the plasma membrane [16,20]. In addition, Vpu can remove tetherin from the plasma membrane [16,20,21], although with relatively low efficiency, and can induce tetherin ubiquitination and degradation in endolysosomes [22,23,24]. However, the contribution of these processes to tetherin antagonism by Vpu seems to be minor. Finally, it is notable that species specific differences in the amino acid sequence of the tetherin transmembrane domain can alter susceptibility to antagonism by Vpu [11], underlining that the tetherin interaction with Vpu is highly specific. 

The glycoproteins (GP) of ebolaviruses and marburgviruses, members of the *Filoviridae* family and highly pathogenic to humans, also antagonize tetherin, allowing efficient release of retro- and filovirus-like particles from tetherin transfected cells [25,26]. In addition, the tetherin antagonism by GP is likely responsible for the very modest [26] or absent [27] inhibition of release of authentic Ebola virus (EBOV) by tetherin. Notable differences between tetherin antagonism by Vpu and EBOV-GP have been reported. A direct interaction of the GP2 subunit of EBOV-GP and tetherin has been documented [26] but might be dispensable for tetherin antagonism, since EBOV-GP unlike Vpu can counteract artificial tetherin [28]. Similarly, EBOV-GP but not Vpu antagonizes tetherin orthologues from different mammalian species [26]. Moreover, EBOV-GP in contrast to Vpu does not reduce tetherin expression at the cell surface [26,28] and does not remove tetherin from lipid rafts [28,29]. In sum, EBOV-GP and Vpu employ markedly different mechanisms to counteract tetherin and the determinants in GP, which govern tetherin counteraction, are largely unclear.

Lassa virus (LASV), a member of the *Arenaviridae* family and like EBOV highly pathogenic to humans, is inhibited by tetherin and the viral glycoprotein (GPC) does not antagonize tetherin [27,30]. A previous study constructed chimeras between the Marburg virus GP (MARV-GP) and LASV-GPC in order to identify determinants of viral assembly [31]. Here, we employed these LASV-GPC/MARV-GP chimeras and newly constructed LASV-GPC/EBOV-GP chimeras to map domains important for tetherin counteraction. We found that the cytoplasmic tail of EBOV- and MARV-GP and the integrity of the fusion peptide sequence in EBOV-GP are dispensable for tetherin counteraction. The exchange of the transmembrane or cytoplasmic domains between EBOV-GP/MARV-GP and LASV-GPC impeded tetherin counteraction by the filovirus GPs and did not transfer tetherin antagonism to LASV-GPC, indicating that the determinants in GP, which govern tetherin antagonism, are complex.

## 2. Results

### 2.1. LASV-GPC Fails to Antagonize Tetherin in the Context of a HIV-1 Gag-Based Virus-Like Particle System

**Figure 1:**
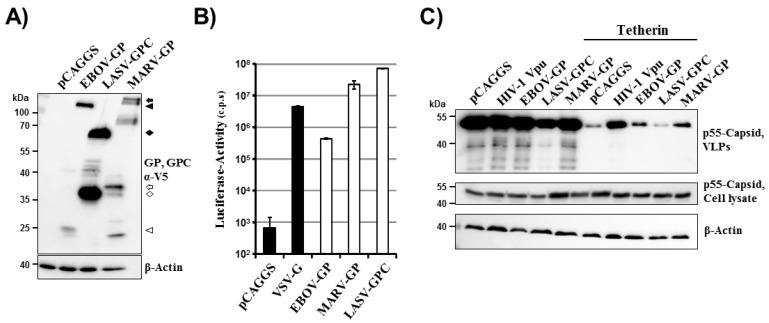
Filovirus but not Lassa virus glycoproteins counteract tetherin. (**A**) Plasmids encoding the indicated glycoproteins with a C-terminal V5 tag were transfected in 293T cells and GP expression was analyzed by Western blot employing a V5-specific antibody. Expression of β-actin in cell lysates was determined as loading control. Similar results were obtained in at least four separate experiments. The V5-specific antibody allowed detection of glycoprotein precursors (EBOV-GP0: black triangle; MARV-GP0: black arrow; LASV-GPC: black diamond) and their mature transmembrane units (EBOV-GP0: white triangle; MARV-GP2: white arrow, LASV-GP2: white diamond), which are generated upon cleavage of the precursor proteins by host cell proteases. Additional bands most likely represent processing intermediates and different glycoforms. (**B**) HIV-1-derived pseudotypes bearing the indicated GPs (without V5 tag) were used to transduce 293T target cells. Three days after transduction, the GP-mediated cell entry was quantified by determining luciferase activity in cell lysates (c.p.s., counts per second). The results of a single experiment are shown; error bars indicate standard deviation (SD). The results were confirmed in two separate experiments. (**C**) 293T cells were co-transfected with an expression plasmid encoding HIV-1 p55 Gag, empty plasmid (pCAGGS) or tetherin encoding plasmid jointly with plasmids encoding Vpu or the indicated glycoproteins. Subsequently, pelleted virus-like particles (VLPs) and cell lysates were analyzed for HIV-1 Gag content by Western blot. Expression of β-actin in cell lysates was determined as loading control. Similar results were obtained in three separate experiments.

We sought to employ chimeric EBOV/MARV and LASV glycoproteins to identify domains important for tetherin counteraction. As a prerequisite to these studies, we first determined tetherin counteraction by the wt proteins. Expression of EBOV-GP, MARV-GP and LASV-GPC with a C-terminal V5 antigenic tag was readily detectable by Western blot analysis of transfected 293T cells (Figure 1A). As expected, all precursor glycoproteins and their mature C-terminal transmembrane units, which are generated upon processing of the precursors by host cell proteases, were detected and largely exhibited the expected molecular weights [32,33,34,35,36,37]. Moreover, a lentiviral vector pseudotyped with these glycoproteins (without V5 tag) was able to efficiently transduce 293T cells while no appreciable transduction was observed upon inoculation of cells with a vector bearing no glycoprotein (Figure 1B). Thus, the constructs available for our study allowed for robust glycoprotein expression and the glycoproteins were able to mediate efficient host cell entry, as expected. In order to determine whether the glycoproteins were able to counteract tetherin, a previously described virus-like particle system was employed [26]. This system is based on HIV-1 p55 Gag, which is sufficient to drive budding, and budding can be inhibited by tetherin. LASV-GPC has previously not been characterized in this experimental setup. Efficient release of Gag into the supernatants of cultured cells was observed and, in the absence of tetherin, release was not modulated by coexpression of HIV-1 Vpu, EBOV-GP, MARV-GP or LASV-GPC (Figure 1C), in concordance with published data [26]. In contrast, coexpression of tetherin markedly reduced Gag release and this effect could be partially rescued by expression of Vpu, EBOV-GP and MARV-GP but not LASV-GPC (Figure 1C), again in keeping with previous studies [25,26,27]. Thus, filoviral GPs but not LASV-GPC function as tetherin antagonists in the context of HIV-1 Gag-dependent budding, and mutagenic analysis of these glycoproteins might reveal domains required for tetherin antagonism.

### 2.2. The Cytoplasmic Tail of EBOV-GP and MARV-GP Is Dispensable for Tetherin Counteraction

**Figure 2:**
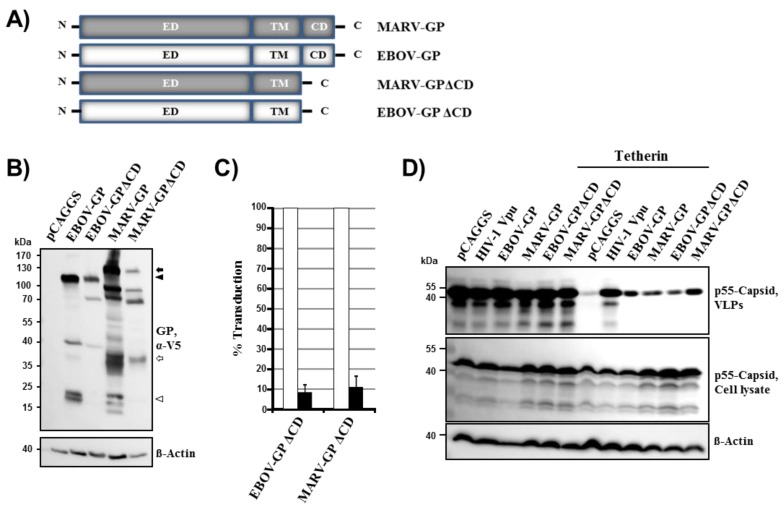
The cytoplasmic tail of filoviral glycoproteins is dispensable for tetherin antagonism. (**A**) Schematic representation of GP mutants analyzed. ED, extracellular domain; TM, transmembrane domain; CD, cytoplasmic domain. (**B**) The indicated glycoproteins harboring a C-terminal V5 antigenic tag were transiently expressed in 293T cells and expression analyzed by Western blot (upper panel), as described for Figure 1A. Expression of β-actin in cell lysates was determined as loading control (lower panel). Similar results were obtained in two separate experiments. Bands corresponding to glycoprotein precursors and their mature, transmembrane units are marked as described for Figure 1A. (**C**) Transduction of target cells by wt and mutant GPs was determined as described for Figure 1B. The average ± standard error of the mean (SEM) of at least three separate experiments is shown. Signals measured for wt GPs were set as 100%. (**D**) Tetherin antagonism by filoviral glycoproteins lacking the cytoplasmic domain was analyzed as described for Figure 1C. The results were confirmed in three separate experiments.

The cytoplasmic tails of EBOV-GP and MARV-GP comprise four and eight amino acids, respectively, and have previously been shown to be important for robust GP-driven virus-cell fusion [38]. Their role in tetherin antagonism by GP has not been determined. Expression of EBOV-GP and MARV-GP mutants lacking the cytoplasmic tail, EBOV-GPΔCD and MARV-GPΔCD (Figure 2A), was readily detectable with antibody specific for the V5 tag, although somewhat less efficient than expression of the wt proteins (Figure 2B). Similarly, mutant EBOV-GPΔCD was incorporated into virus-like particles (VLPs) although with slightly reduced efficiency compared to its wt counterpart (not shown). Lentiviral vectors bearing EBOV-GPΔCD or MARV-GPΔCD (without V5 tag) showed a markedly reduced capacity to transduce 293T cells when compared to vectors equipped with the wt protein (Figure 2C), in keeping with the documented importance of the cytoplasmic tail for GP-driven entry [38]. Despite their modestly reduced expression, both EBOV-GPΔCD and MARV-GPΔCD were readily able to counteract tetherin (Figure 2D), indicating that the cytoplasmic tail is not required for tetherin antagonism by filoviral glycoproteins.

### 2.3. The Transmembrane Domain in EBOV-GP and MARV-GP Is Essential for Virus-Cell Fusion and for Tetherin Antagonism

**Figure 3:**
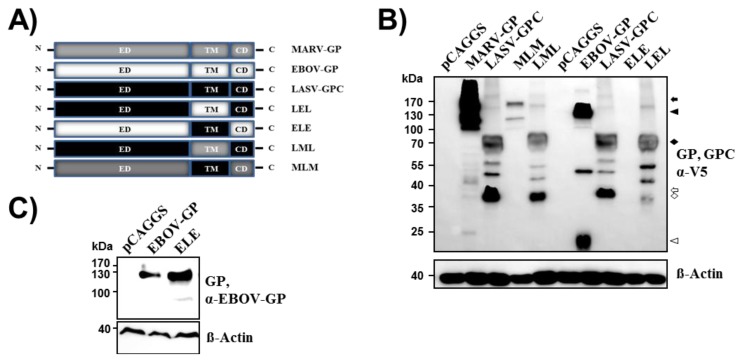
The exchange of the transmembrane domains between filoviral and Lassa virus glycoprotein is compatible with robust expression. (**A**) Schematic overview of the chimeric glycoproteins analyzed. (**B**) Expression of V5-tagged glycoprotein chimeras was tested as described for Figure 1A. Expression of β-actin in cell lysates was determined as loading control. Bands corresponding to glycoprotein precursors and their mature, transmembrane units are marked as described for Figure 1A. (**C**) Expression of EBOV-GP wt and mutant ELE in the cell lysates analyzed in (**B**) was determined employing an antibody raised against EBOV-GP. Signals correspond to the surface unit GP1. Expression of β-actin in cell lysates was determined as loading control. The results were confirmed in at least two separate experiments.

We next examined the role of the transmembrane domain of filovirus GPs in tetherin counteraction. Western blot analyses with anti-V5 antibody revealed that the exchange of the transmembrane domain between EBOV-GP/MARV-GP and LASV-GPC (Figure 3A) was compatible with robust expression (Figure 3B), in keeping with published data [31], although expression of MLM was reduced compared to expression of wt MARV-GP. Weak or no expression (Figure 3B and not shown) of mutant ELE was observed upon detection with an antibody directed against the V5 tag. However, a signal comparable to that detected for wt EBOV-GP was observed when expression of ELE with V5 tag was visualized with an EBOV-GP specific antibody (Figure 3C), suggesting that this mutant was readily expressed. 

**Figure 4:**
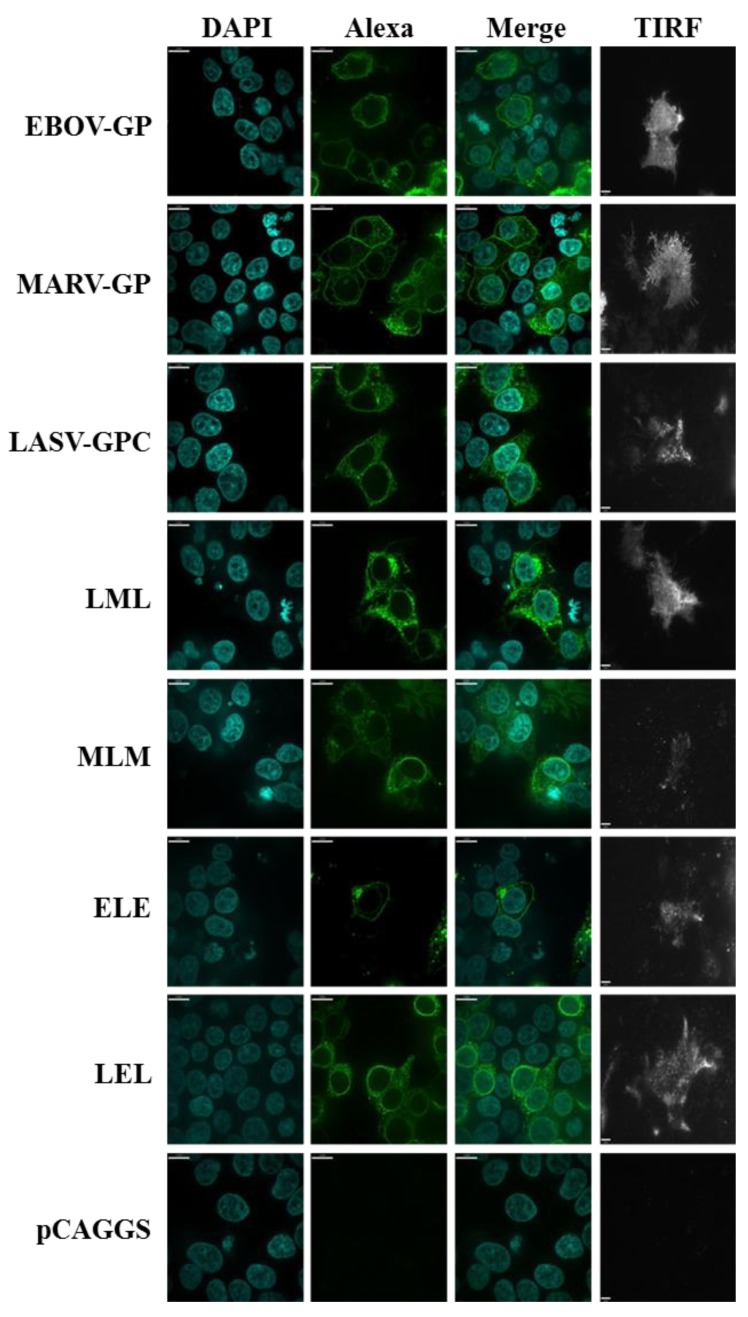
Cellular localization of filoviral and Lassa virus glycoproteins with exchanged transmembrane domains. The indicated glycoproteins were transiently expressed in 293T cells and GP expression was detected by staining with V5-specific antibody and Alexa Fluor 488 labeled secondary antibody. Nuclei were visualized by DAPI staining. Staining was analyzed by spinning disc microscopy. In addition, surface localized GP was detected by TIRF microscopy.

In order to analyze whether the exchange of the transmembrane domain modulated the cellular localization of the glycoprotein variants studied, we employed spinning disk confocal microscopy and total internal reflection fluorescence (TIRF) microscopy. Staining of cells with anti-V5 antibody and analysis by confocal microscopy revealed no major changes between the localization of mutants LML, ELE and LEL and the respective wt proteins, although a relatively low percentage of cells were positive for mutant ELE (Figure 4). Moreover, TIRF microscopy showed that both wt proteins and mutants LML, ELE and LEL were appreciably expressed at the plasma membrane (Figure 4). In contrast, mutant MLM was predominantly localized within the cells, although some plasma membrane localization was detected. In keeping with the expression of ELE, LEL, LML, and to a lesser degree MLM at the plasma membrane, all mutants were efficiently incorporated into VLPs (no shown). Thus, the exchange of the transmembrane domain between EBOV-/MARV-GP and LASV-GPC is largely compatible with glycoprotein expression at the cell surface and particle incorporation.

**Figure 5:**
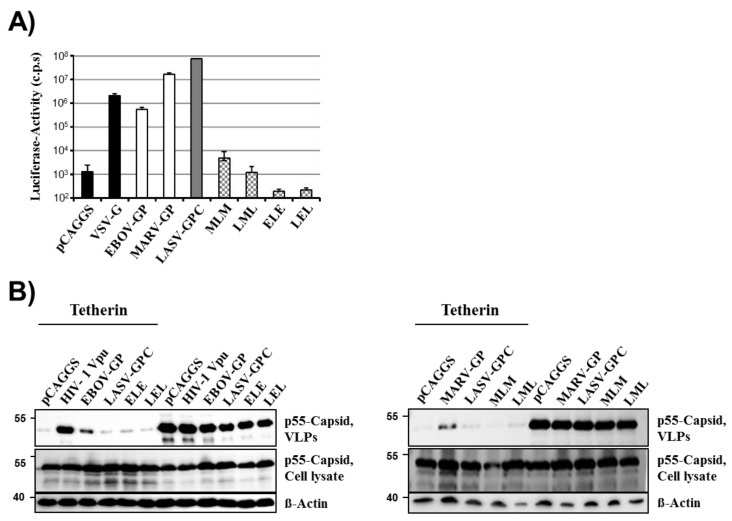
The filoviral transmembrane domain is necessary but not sufficient for tetherin antagonism. (**A**) Transduction of cells with pseudotypes bearing the indicated chimeric GPs was analyzed as described in Figure 1B. One representative experiment is displayed. Results were confirmed in at least five separate experiments. Error bars indicate standard deviation. (**B**) Tetherin antagonism of the indicated chimeric GPs was analyzed as described in Figure 1C. The results of a representative experiment are shown and were confirmed in at least four additional experiments.

Despite readily detectable expression and particle incorporation of all glycoprotein mutants with exchanged transmembrane domain, none of the mutants (without V5 tag) was able to appreciably drive virus-cell fusion (Figure 5A), indicating that the appropriate transmembrane domain is essential for GP function in viral entry. Similarly, none of the transmembrane domain mutants was able to counteract tetherin. Thus, the introduction of the transmembrane domain of EBOV-GP and MARV-GP into LASV-GPC did not confer tetherin antagonism to LASV-GPC while the reverse exchange abrogated tetherin counteraction by EBOV-GP and MARV-GP (Figure 5B). These results indicate that the transmembrane domain of the glycoproteins studied is essential for their ability to mediate virus-cell fusion. Moreover, our findings show that the transmembrane domain of EBOV-GP and MARV-GP is required for tetherin counteraction but is not sufficient to confer tetherin antagonism to a heterologous glycoprotein.

### 2.4. Conserved Residues in the Fusion Peptide and Transmembrane Domain of EBOV-GP Are Dispensable for Tetherin Counteraction

**Figure 6:**
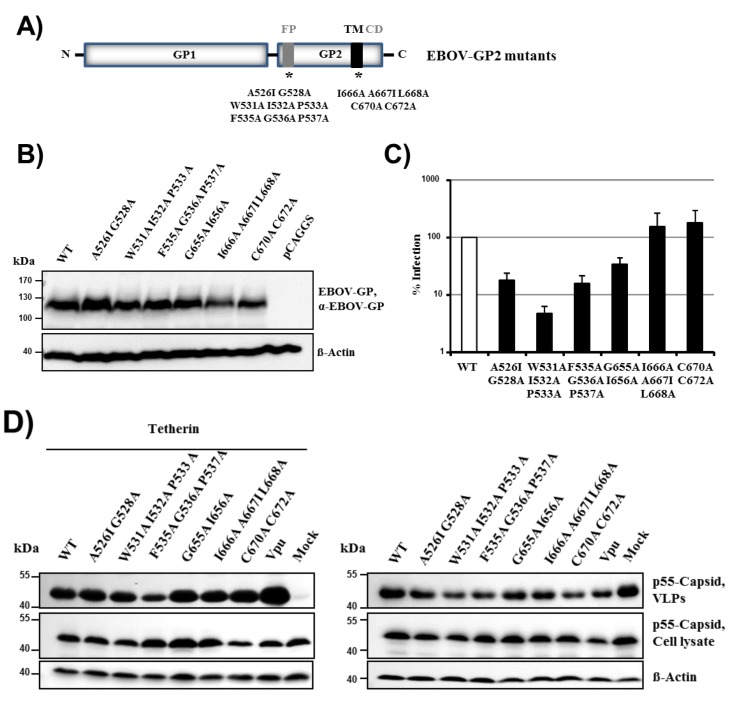
Conserved residues in the fusion peptide and transmembrane domain of EBOV-GP are dispensable for tetherin antagonism. (**A**) Overview of the domain organization of EBOV-GP. Domains mutagenized within GP2 are indicated with asterisks. IFL, internal fusion loop; TM, transmembrane domain; CD, cytoplasmic domain. Asterisks indicate positions were mutations were inserted. (**B**) The expression of the GP mutants was analyzed as described for Figure 1A; an antibody raised against EBOV-GP was used for detection. Signals correspond to the surface unit GP1. The expression of β-actin was determined as loading control. The results were confirmed in two separate experiments. (**C**) Cellular entry driven by the GP mutants was assessed as described in the legend to Figure 1B. Transduction mediated by EBOV-GP wt was set as 100%. The average of five individual experiments is shown. Error bars indicate SEM. (**D**) Tetherin antagonism of the EBOV-GP mutants was analyzed as described for Figure 1C. A representative experiment is shown. Similar results were obtained in five separate experiments.

The finding that the transmembrane domain in filovirus GP is essential for tetherin antagonism (Figure 5B) prompted us to investigate whether specific amino acids in the transmembrane domain can be identified, which are required for tetherin counteraction. For this, we mutated residues in the transmembrane domain of EBOV-GP, which are conserved among filovirus GPs (mutants G655A + I656A, I666A + A667I + L668A, C670A + C672A, Figure 6A). Moreover, the observation that all chimeric GPs had lost their ability to drive virus-cell fusion and their capacity to counteract tetherin (Figure 5A,B) suggested that these two activities might depend on the same functional elements in GP. We therefore asked if mutation of conserved residues in the fusion peptide (FP) of EBOV-GP, which block membrane fusion and thus viral entry into target cells, might also interfere with tetherin antagonism (mutants A526I + G528A, W531A + I532A + P533A, F535A + G536A + P537A). All GP mutants were efficiently expressed in 293T cells, although expression of mutants I666A + A667I + L668A, C670A + C672A was slightly reduced compared to expression of EBOV-GP wt (Figure 6B). Similarly, all GP mutants were efficiently incorporated into VLPs (not shown). The mutations in FP invariably reduced the ability of GP to drive virus-cell fusion, in keeping with published data [39], while alteration of the transmembrane domain had little (mutant G655A + I656A) or no inhibitory effect (mutants I666A + A667I + L668A, C670A + C672A) (Figure 6C) Notably, all mutants were able to counteract tetherin (Figure 6D), indicating that neither the capacity to efficiently drive membrane fusion nor the presence of several conserved amino acids in the transmembrane domain are required for tetherin antagonism. 

## 3. Discussion

The filovirus GPs antagonize tetherin in VLP-based systems and likely allow for the spread of authentic filoviruses in tetherin-positive cells [25,26]. However, the domains in GP required for tetherin antagonism remained elusive. Our previous work demonstrated that the GP2 portion of EBOV-GP interacts with tetherin [26]. Here, we analyzed whether domains in GP2, in particular the transmembrane domain and the fusion peptide, contribute to tetherin counteraction by EBOV- and MARV-GP. We show that the presence of the authentic transmembrane domain in EBOV-GP and MARV-GP is required for GP-driven virus-cell fusion and tetherin antagonism. However, exchange of the transmembrane domain was not sufficient to transfer tetherin antagonism to a heterologous viral glycoprotein. In addition, we demonstrate that the capacity of GP to mediate virus-cell fusion is not required for tetherin antagonism. These results suggest that the transmembrane domain contributes to tetherin antagonism by filovirus GPs but that other domains also play a role.

The filoviruses EBOV and MARV induce a severe and frequently lethal hemorrhagic fever in infected human patients and non-human primates [40,41]. The GPs of these viruses mediate entry into host cells and facilitate transduction of a broad range of target cells upon incorporation into retroviral or rhabdoviral vectors [42,43]. In addition, filovirus GPs rescue budding driven by filovirus VP40 or HIV-1 Gag from inhibition by tetherin [25,26] and the relatively robust release of EBOV from tetherin expressing cells [26,27] suggests that GP can also protect authentic filoviruses from tetherin’s antiviral action. In contrast, spread of authentic LASV is inhibited by tetherin and LASV-GPC [27]—like filovirus GPs a class I membrane fusion protein—fails to counteract tetherin in a VLP-based system [27], in keeping with the results obtained in the present study. The domains in filoviral GPs and LASV-GPC responsible for their differential ability to counteract tetherin are unknown. Therefore, domain-swaps between filoviral GPs and LASV-GPC as well as alteration of functionally important domains in GPs could be suitable ways to detect determinants in GP, which control tetherin antagonism.

We first assessed whether the short cytoplasmic tail of EBOV-GP and MARV-GP is required for tetherin counteraction. It has previously been documented that the cytoplasmic tail is dispensable for GP expression, trimerization, intracellular transport or incorporation into filovirus-like particles [38]. However, deletion of the cytoplasmic tail altered GP glycosylation and recognition by neutralizing antibodies and impaired host cell entry of filovirus-like particles [38]. Our results confirm that deletion of the cytoplasmic tail is dispensable for robust GP expression and particle incorporation (not shown) but impedes efficient GP-driven virus-cell fusion. The cytoplasmic tail was dispensable for tetherin counteraction, in keeping with previous results showing that amino acids in the cytoplasmic tail believed to be important for lipid raft localization of GP are dispensable for tetherin counteraction [44]. Thus, the cytoplasmic tail of GP seems to be required for GP function during viral entry but is dispensable for counteraction of tetherin during viral release.

The analysis of chimeras between LASV-GPC and MARV-GP previously demonstrated that the presence of the authentic transmembrane domain in MARV-GP is required for transport of GP into multi-vesicular bodies containing elevated levels of the viral matrix protein VP40 and for particle incorporation of GP [31]. Our results confirm the previously documented observation that exchange of the transmembrane domain between MARV-GP and LASV-GP is compatible with robust expression of the resulting chimeras [31], although reduced levels of mutant MLM relative to MARV-GP wt were detected in cells (and might partially account for the lack of tetherin counteraction by this mutant), and we show that the same applies to EBOV-GP. In addition, our results indicate that exchange of the transmembrane domain does not interfere with GP localization at the cell membrane, the site where tetherin unfolds its antiviral activity, with mutant MLM being the only exception. Nevertheless, all chimeric GPs failed to mediate virus-cell fusion, despite efficient incorporation into lentiviral vectors (data not shown). Similarly, all chimeric proteins, including LASV-GPC variants harboring a filovirus GP-derived transmembrane domain, failed to counteract tetherin. In contrast, residues within the EBOV-GP transmembrane domain, which are conserved among all filovirus GPs, were dispensable for tetherin counteraction. These findings indicate that the transmembrane domain contributes to but is not the exclusive determinant of tetherin counteraction. Moreover, they suggest that the role of the transmembrane domain in tetherin antagonism by filoviral GPs might be due to its potential impact on GP conformation or cellular localization rather than the presence of specific amino acids within this domain.

The GP2 subunit of the filovirus GP contains the functional elements, which drive virus-cell fusion, including a fusion peptide. This peptide inserts into the target cell membrane during virus-cell fusion and its integrity is essential for viral infectivity [39]. The analysis of chimeric GPs suggested that the capacity of GP to drive efficient host cell entry might be a prerequisite to tetherin counteraction while the opposite conclusion could be drawn from the characterization of GP mutants without cytoplasmic tail. In order to assess whether the ability to mediate virus-cell fusion in general and the integrity of the fusion peptide in particular are important for tetherin counteraction, we analyzed EBOV-GP mutants with amino acid changes in the fusion peptide. The respective GP mutants were robustly expressed but failed to drive efficient virus-cell fusion. This observation matches results obtained by a previous study, which demonstrated that exchanges in the fusion peptide are compatible with robust virion incorporation of GP but not with GP-driven virus-cell fusion [39]. In particular, single mutants F535A, G536A and P537A (triple mutant F535A + G536A + P537A was analyzed in the present study) were efficiently incorporated into a VSV vector but failed to drive efficient virus cell fusion [39]. All fusion peptide mutants tested were able to counteract tetherin, indicating that the integrity of the fusion peptide and the ability of GP to efficiently drive virus-cell fusion is dispensable for tetherin counteraction.

## 4. Experimental Section

### 4.1. Cell Culture and Transfection

Human embryonic kidney 293T cells were maintained in Dulbecco’s modified Eagle’s medium (DMEM) supplemented with 10% fetal calf serum (FCS) and antibiotics. Cells were grown at 37 °C and 5% CO_2_. The day prior transfection cells were seeded at a density of 2.8 × 10^5^ cells/6 well format or 7 × 10^5^ cells/T25 flasks. Cells were transfected by the calcium phosphate method using 6 µg DNA in total for one well of a 6-well plate or 12 µg DNA in total for transfection of a T25 flask. Medium was exchanged eight to 16 h post transfection. 

### 4.2. Plasmids

Plasmids encoding HIV-1 Gag (p55) [45], Vpu [46], LASV-GPC [47], EBOV-GP [48], EBOV-GP with C-terminal V5-tag [49], MARV-GP [50], human tetherin [51] and HIV-1 NL4-3 derived vector pNL4-3 E^–^R^–^Luc [52] were described previously. Plasmids encoding chimeras between LASV-GPC and MARV-GP in which the transmembrane domain was exchanged (mutants LML, MLM) and EBOV-GP and MARV-GP with a deleted cytoplasmic domain (CD) (mutants MARV-GPΔCD, EBOV-GPΔCD) were also described previously [31,38]. Additional chimeric GPs in which the transmembrane domain was exchanged between LASV-GPC and EBOV-GP (mutants ELE, LEL) as well as glycoproteins with a C-terminal V5 tag were generated by PCR. All PCR products were inserted into pCAGGS expression vector [53] (restriction sites: Lassa-GPC-V5 and MARV-GP-V5: EcoRI, XhoI; MLM-V5 and MARV-GPΔCD-V5: EcoRI, NheI; LML-V5, ELE, ELE-V5, LEL, LEL-V5 and EBOV-GPΔCD-V5: XmaI, NheI). EBOV-GP mutants with point mutations in FP and TM were also generated by PCR-based mutagenesis and inserted via XbaI, MluI into pCAGGS. The integrity of all PCR-amplified sequences was analyzed by automated sequencing.

### 4.3. Expression Analysis of Glycoproteins

To test the expression levels of the glycoproteins analyzed, 6 µg of the respective expression plasmids were transfected into 293T cells seeded in 6-well plates. At 48 h post transfection, the cells were harvested, lysed and glycoprotein expression in cell lysates detected by Western blot. For detection, a mouse anti V5 antibody (Invitrogen, Carlsbad, CA, USA) was used at a 1:2500 dilution. Alternatively, a mouse anti-EBOV-GP antibody was used (3B11, [54]) at a dilution of 1:1000.

### 4.4. Inhibition of Virus-Like Particle Release by Tetherin

In order to analyze tetherin antagonism, a previously described virus-like particle system was used [26]. 293T cells seeded in 6-well plates were cotransfected with plasmids encoding Gag, tetherin, and the respective tetherin antagonist in a ratio of 2:1:1. Forty-eight hours after transfection, supernatants were cleared from debris at 4000 rpm and pelleted for 2 h through a 20% sucrose cushion at 13,000 rpm. The pelleted VLPs as well as the producer cells were lysed and boiled in SDS-loading buffer and subsequently analyzed by Western blot. The Gag protein was detected with an anti-p24 hybridoma supernatant (183-H12-5C, 1:500) and a goat anti mouse-HRP antibody (Dianova, Hamburg, Germany; 1:1000) using a commercially available kit (GE Healthcare, Chalfont St Giles, UK). 

### 4.5. Production of Pseudotypes and Transduction Experiments

For generation of HIV-1 NL4-3-based pseudotypes carrying the different glycoproteins including VSV-G, filoviral GPs, LASV-GPC, chimeric GPs or no glycoprotein as control, a previously described protocol was used [47]. Briefly, cells seeded in T25 flasks were transfected with pNL4-3 E^–^R^–^Luc and glycoprotein expression plasmid at a 1:1 ratio. At 48 h post transfection, supernatants were harvested and passed through 0.45 μm pore-size filters. For subsequent transduction, 293T cells were seeded in 96-well plates at a density of 5 × 10^4^ cells/well. Cells were then transduced with 50 μL of vector containing supernatants. Medium was exchanged 8 h post transduction and transduction efficiency was measured by determining luciferase activity in cell lysates at 72 h post infection using a commercially available kit (Promega, Madison, WI, USA).

### 4.6. Immunofluorescence, Spinning Disc Confocal Microscopy and Total Internal Reflection Fluorescence (TIRF) Microscopy

4 × 10^5^ 293T cells were seeded in 22 mm glass bottom culture dishes (WillCo-dish, Amsterdam, The Netherlands) and calcium phosphate transfected with 3 µg of V5-tagged glycoprotein expression plasmids or the empty plasmid as control. At 24 h post transfection, cells were fixed with 2% PFA for 20 min at 4 °C and permeabilized with 1 % Saponin for 10 min at RT. Subsequently, cells were stained for V5 using a 1:200 dilution of anti-V5 specific antibody (Invitrogen) and 1:500 diluted goat anti-mouse Alexa Fluor 488 conjugated secondary antibody (Molecular Probes, Invitrogen). TIRF microscopy was conducted with a Nikon TiE microscope using the 100× objective (NA 1.49) essentially as previously described [55]. For confocal imaging, the same samples which were transfected and stained for TIRF were mounted with DAPI containing Mowiol. Spinning disc confocal microscopy was done with a Nikon TiE equipped with the PerkinElmer UltraView Vox system. Images were analyzed with the Volocity 6.3-software package (Perkin Elmer, Waltham, MA, USA) [56]. 

## 5. Conclusions

Our results suggest that several determinants in filovirus GPs might control tetherin counteraction. Future studies must address whether functional domains in the GP1 subunit, like the receptor binding domain, which mediates interactions with NPC-1 [57,58], play a role in tetherin counteraction. Similarly, it will be interesting to investigate why EBOV-GP, although able to counteract wt and artificial tetherin [28], fails to enhance release of LASV-like particles [27]—an observation highlighting that the determinants which govern tetherin inhibition of viral release and its counteraction by filoviral glycoproteins are complex.
